# Platelet counts affect the association between hyperhomocysteinemia and pregnancy complications

**DOI:** 10.1186/s12889-023-16027-6

**Published:** 2023-06-02

**Authors:** Bin Yu, Bin Zhang, Xiaoya Han, Wei Long, Wenbo Zhou, Xiaosong Yuan

**Affiliations:** Department of Medical Genetics, Changzhou Maternal and Child Health Care Hospital, Changzhou Medical Center, Nanjing Medical University, 16th Ding Xiang Road, Changzhou, 213023 Jiangsu China

**Keywords:** Homocysteine, Platelet, Pregnancy complications, Intrahepatic cholestasis of pregnancy, Pregnant women, Gestational diabetes mellitus

## Abstract

**Background:**

The joint effect of platelet and other modifiers on the risk of pregnancy complications is unknown. This study investigated whether platelet count (PC) and total homocysteine (tHcy) level have a synergistic effect on the incidence of pregnancy complications in a Chinese population.

**Methods:**

Total 11,553 consecutive pregnant women who received whole blood cell and biochemical tests at the time of admission for labor in Changzhou Maternal and Child Health Care Hospital were analyzed. The primary outcome was the prevalence of pregnancy complications: gestational diabetes mellitus (GDM), intrahepatic cholestasis of pregnancy (ICP), pre-eclampsia (PE), and pregnancy induced hypertension (PIH).

**Results:**

The prevalence of GDM, ICP, PE, and PIH was 8.4%, 6.2%, 3.4%, and 2.1%, respectively. The highest rate of ICP (28.6%) was observed in women with high tHcy (> 15 μmol/L) and low PC (quartile 1); and the lowest rate of GDM (0.6%) was found in women with high tHcy and high PC (quartiles 2 to 4). In low PC group, the prevalence of ICP in women with high tHcy was significantly higher than that in women with low tHcy (≤ 15 μmol/L) (28.6% vs. 8.4%), representing an absolute risk increment of 20.2% and a relative risk increment of 3.3-fold (OR: 3.34; 95% CI: 1.55, 7.17; *P* = 0.002), whereas no joint effect was observed among high PC group.

**Conclusions:**

Among Chinese pregnant women, one subgroup (high tHcy and low PC) has the highest risk of ICP and another (high tHcy and high PC) has the lowest risk of GDM; tHcy and platelet could be used as indicators to identify the women with high risk of ICP or low risk of GDM.

## Background

Pregnancy complications, such as gestational diabetes mellitus (GDM), intrahepatic cholestasis of pregnancy (ICP), pre-eclampsia (PE), and pregnancy induced hypertension (PIH), affect more than one fifth of all pregnancies and jeopardize the health of mothers and their infants in a short and/or long term besides adverse perinatal outcomes [1]. GDM complicates 7–13% of pregnancies and women who experienced GDM have a 32% increased risk of venous thrombosis, a 69% increased risk of cardiovascular disease (CVD), a 89% increased risk of hypertension, a 2.1-fold increase in risk of cardiovascular and metabolic morbidity, a 4.5-fold increase in dyslipidemia risk, and a nearly tenfold higher risk of type 2 diabetes mellitus when compared to those with a normoglycemic pregnancy [2–5]. The offspring of mothers with GDM appear to be at a greater risk of developing overweight in adolescence; female infants born to the affected mothers are particularly likely to develop GDM during their pregnancies [6, 7]. ICP occurs in 6% of pregnancies, which has been associated with adverse birth outcomes for the fetus and subsequent hepatobiliary disease for the mothers [8, 9]. PE affects 2–8% of pregnancies and causes a significant proportion of maternal and neonatal morbidity and mortality [10]. Women with a history of PE have a 2.5-fold increase in risk of coronary heart disease (CHD) and a 4.2-fold increase in risk of incident heart failure [11]. PIH complicates 1–6% of pregnancies and contributes to a greater risk of CVD, CHD, and heart failure by 81%, 83%, and 71%, respectively [12]. Therefore, targeted screening and risk-reduction strategies for women at high risk of pregnancy complications may help decrease the harm to mothers and their infants.

Platelets undergo physiological changes in number and function during pregnancy. While an important role for platelet in the pathogenesis of CVD among the general population has been demonstrated, evidence is conflicting regarding the associations of pregnancy complications with maternal platelet quantity and function. For example, two studies from China and Iran reported that higher platelet count (PC) at 4–20 and 24–28 weeks of gestation was an independent predictor of GDM [13, 14]. Another two studies from China and Turkey showed that PC at 11–13 and 24–28 weeks of gestation in GDM group was significantly higher than that in non-GDM group, but was not independently associated with GDM [15, 16]. Evidence from other different ethnic studies found no differences in PC between the two groups in the first and second trimesters [17–21]. Similar to the case of GDM, the association and predictive efficiency between PC and PE/PIH have also been greatly inconsistent in different studies [22, 23]. In addition, a limited number of case–control studies investigated platelet indices in ICP patients and controls, and observed elevated mean platelet volume (MPV) values rather than PC in those with ICP [24–27].

A number of studies have reported that high total homocysteine (tHcy) was associated with multiple pregnancy complications, including PE, GDM, placental abruption, recurrent pregnancy loss, preterm delivery, and fetal growth restriction. However, findings from these studies also lack consistency [28]. And no studies have investigated the effects of high tHcy on ICP prevalence. It is plausible that tHcy and platelets might mutually enhance and thereby jointly affect the incidence of pregnancy complications, since they are independent risk factors of endothelial injury involved in pathogenesis of these complications [29, 30]. However, to date, little attention has been paid to evaluate the joint association of platelet and tHcy with the incidence of pregnancy complications. Therefore, large observational cohort studies focusing on the impacts of platelet and tHcy on pregnancy complications are urgently needed. The aim of this study was to examine whether an elevated frequency of pregnancy complications was evident among women with low platelet counts and high tHcy levels in a homogeneous population.

## Materials and methods

### Study design and data collection

An observational cohort study was conducted on 13,275 consecutive pregnant women who delivered at Changzhou Maternal and Child Health Care Hospital (a 3-A-Class Specialized Hospital) between 2016 and 2017. Women who fulfilled the following criteria were included: detailed medical records, singleton pregnancy, live birth with no birth defects, and complete laboratory tests. Women were excluded if they met these conditions: smoking or use of alcohol and illicit drugs during pregnancy; a history of pre-pregnancy diseases that affect the PC, tHcy level and pregnancy outcomes, including diabetes mellitus (type 1 or 2), chronic hypertension, chronic heart, liver and kidney diseases, immune rheumatic disease, syphilis, and thrombocytopenia. Among the 13,275 initial subjects to observe, 1722 pregnant women who presented with pre-pregnant diseases (*n* = 488), multiple gestation (*n* = 335), absence of live birth (*n* = 96), without platelet count and tHcy level (*n* = 803) were excluded from final analysis. Baseline data and pregnancy outcomes were downloaded from electronic medical record of the hospital, including maternal age, height, body weight, gravidity, parity, blood pressure, use of illicit drugs and alcohol, medical history, pregnancy complications, neonatal gender, height, body weight, and gestational age. None of the women smoked, drank alcohol, and used illegal drugs during pregnancy. At the time of admission to the hospital, blood samples were collected and transferred to the laboratory for whole blood cell analysis and biochemical test including hepatic and renal function, blood lipid, high sensitive C-reactive protein (hs-CRP), folic acid, vitamin B12, and tHcy. The results of these tests were obtained from the laboratory database. In this study, hepatic and renal function, blood lipids, hsCRP, tHcy, folic acid, and vitamin B12 levels were determined by specific automated analyzers with matching reagents, respectively (for hepatic, renal function, and blood lipids: AU5800, Beckman Coulter Inc., Japan; for hsCRP and tHcy: BN II System, Siemens Diagnostics Inc., Germany; for folic acid and vitamin B12: UniCel DxI 800 Access, Beckman Coulter Inc., USA). The whole blood cell counts were analyzed by hematology analyzer (XN550, Sysmex INC., Japan). Inter- and intra-assay coefficient of variation (CV) values for the tests in the laboratory were as follows: <2%/<2% for red blood cell (RBC), <2%/<5% for white blood cell (WBC), <5%/<8% for platelet, and <5%/<10% for tHcy, hs-CRP, folic acid, vitamin B12, hepatic and renal function, and blood lipids.

### Definitions

Advance age, overweight, and obesity were defined as an age ≥ 35 years, a BMI ≥ 25 and < 30 kg/m^2^, and a BMI ≥ 30 kg/m^2^, respectively [3, 31]. Hyperhomocysteinemia, folic acid deficiency, and vitamin B12 insufficiency were defined as a tHcy > 15 μmol/L, a folic acid < 10 nmol/L, and a vitamin B12 < 148 pmol/L, respectively [32, 33]. SGA/AGA/LGA were defined as a birthweight < the 10^th^ percentile, a birthweight ≥ the 10^th^ and ≤ 90^th^ percentiles, and a birthweight > the 90th percentile of gestational age specific cutoff value from the cohort, respectively [1]. PTB was diagnosed as a birth at < 37 gestational weeks [34].

As detailed from a previous study, the primary outcome to observe was pregnancy complications occurring at the time of admission for labor, including GDM, ICP, PE, and PIH [35]. All cases of pregnancy complications were adjudicated by experts in obstetrics according to a previous report [36].

### Statistical analysis

Data are described as frequency (%) for categorical variables and as mean ± standard deviation (SD) for continuous variables by platelet count quartiles and two categories of tHcy levels. The odds ratios (ORs) and 95% confidence intervals (CIs) for pregnancy complications associated with PC and tHcy level were calculated using logistic regression models by adjusting for pertinent confounding factors, including maternal age, BMI, gravidity, parity, assisted reproduction, neonatal sex, and laboratory results. Similarly, the odds ratios (ORs) and 95% confidence intervals (CIs) for the specific complications across each subgroup defined by PC and tHcy level were assessed and their interactions were evaluated. Additionally, the potential effect modifications on the association between hyperhomocysteinemia and ICP due to different subgroups defined by maternal characteristics and their interactions were estimated. Data analysis was carried out using Empower software (X&Y Solutions, Inc. Boston, Massachusetts) and R statistical package (http://www.R-project.org). A *P* < 0.05 was denoted to be statistical significance in the analysis.

## Results

### Characteristics of the study population

**Fig. 1:**
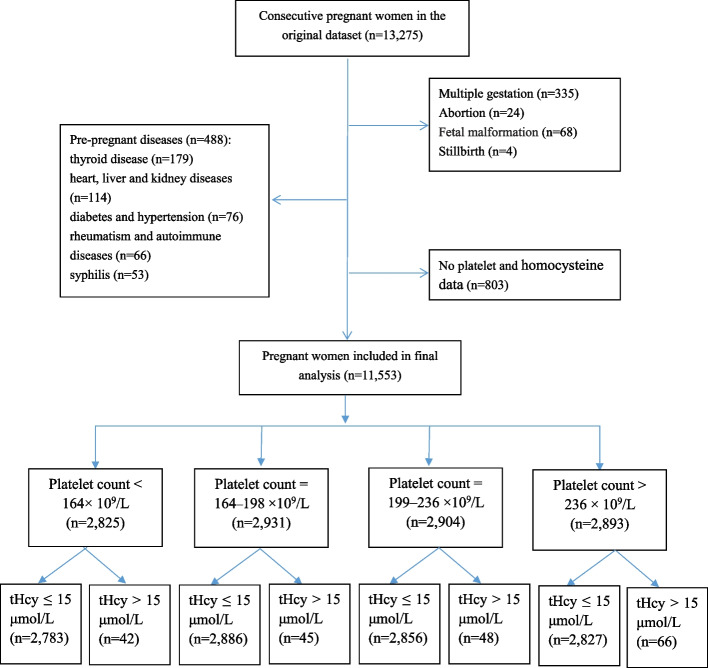
Flow diagram

The flow diagram of the subject to observe is presented in Fig. 1. Of the 13,275 consecutive pregnant women in the initial dataset, the final analyses were limited to 11,553 participants with the measurement of PC and tHcy level at the time of admission for labor. Of these, 1.7% (201/11,553) were defined as hyperhomocysteinemia. The prevalence of GDM, ICP, PE, and PIH in the study population was 8.4% (965/11,553), 6.2% (711/11,553), 3.4% (394/11,553), and 2.1% (245/11,553), respectively.

As listed in Table 1, the participants’ demographic characteristics and laboratory data are shown by maternal PC quartiles (Q): Q1: < 164 × 10^9^/L; Q2: ≥ 164 to ≤ 198 × 10^9^/L; Q3: ≥ 199 to ≤ 236 × 10^9^/L; and Q4: > 236 × 10^9^/L. Except for a step-wise increase in BMI, there were significant decrement trends in maternal age, the prevalence of GDM and ICP, ALT and AST levels from PC Q1 to Q4. With increasing quartile of PC, MPV and the levels of vitamin B12, folic acid, total bilirubin and creatinine increased significantly, while WBC count and hs-CRP level decreased. Significant differences without stable trends were found for systolic BP, the prevalence of PE and PTB, RBC count, and the levels of tHcy, total bile acid (TBA) total bilirubin, direct bilirubin, urea nitrogen, total cholesterol, and LDL-C between platelet count quartiles. When compared to the non-hyperhomocysteinemia group (tHcy ≤ 15 μmol/L), diastolic BP, the prevalence of ICP, PE, and PTB, platelet count, the levels of TBA, direct bilirubin, urea nitrogen, creatinine, and LDL-C, were significantly higher in the hyperhomocysteinemia group, whereas age, BMI, assisted reproduction rate, GDM prevalence, fetal birth length and weight, RBC count, the levels of vitamin B12, folic acid, hs-CRP, ALT, and HDL-C were significantly lower in the hyperhomocysteinemia group (Table 2).

**Table 1:** The characteristics of study population by the quartiles of maternal platelet count (*n* = 11,553)

	Quartiles of platelet count (10^9^/L)				*P* value
	Q1 (< 164, *n* = 2825)	Q2 (164–198, *n* = 2931)	Q3 (199–236, *n* = 2904)	Q4 (> 236, *n* = 2893)	
Maternal characteristics					
Age (years)	29.1 ± 4.4	28.7 ± 4.4	28.4 ± 4.3	28.1 ± 4.5	< 0.001
< 35	2439 (86.3%)	2586 (88.2%)	2600 (89.5%)	2605 (90.0%)	< 0.001
≥ 35	386 (13.7%)	345 (11.8%)	304 (10.5%)	288 (10.0%)	
BMI (kg/m^2^)^a^	26.9 ± 3.2	27.1 ± 3.3	27.4 ± 3.4	27.8 ± 3.5	< 0.001
< 25	777 (27.8%)	794 (27.3%)	700 (24.4%)	586 (20.5%)	< 0.001
25–29	1545 (55.2%)	1578 (54.3%)	1570 (54.7%)	1589 (55.5%)	
≥ 30	478 (17.1%)	532 (18.3%)	601 (20.9%)	688 (24.0%)	
Gravidity					
< 3	1937 (68.6%)	2092 (71.4%)	2088 (71.9%)	2095 (72.4%)	0.007
≥ 3	888 (31.4%)	839 (28.6%)	816 (28.1%)	798 (27.6%)	
Parity					
No child	1608 (56.9%)	1773 (60.5%)	1753 (60.4%)	1802 (62.3%)	< 0.001
≥ 1 child	1217 (43.1%)	1158 (39.5%)	1151 (39.6%)	1091 (37.7%)	
Gestational age (week)	38.7 ± 1.6	38.7 ± 1.5	38.7 ± 1.7	38.6 ± 1.8	0.184
Systolic BP (mmHg)	120.9 ± 12.2	120.6 ± 12.0	120.9 ± 11.8	121.5 ± 12.1	0.032
Diastolic BP (mmHg)	74.4 ± 8.3	74.4 ± 8.2	74.4 ± 8.1	74.9 ± 8.4	0.095
Assisted reproduction	71 (2.5%)	64 (2.2%)	66 (2.3%)	63 (2.2%)	0.814
Delivery mode					
Vaginal delivery	1663 (58.9%)	1654 (56.4%)	1665 (57.3%)	1660 (57.4%)	0.311
Cesarean section	1163 (41.1%)	1277 (43.6%)	1239 (42.7%)	1233 (42.6%)	
Pregnancy complications					
GDM	295 (10.4%)	252 (8.6%)	225 (7.7%)	193 (6.7%)	< 0.001
ICP	246 (8.7%)	166 (5.7%)	164 (5.6%)	135 (4.7%)	< 0.001
PE	115 (4.1%)	91 (3.1%)	80 (2.8%)	108 (3.7%)	0.026
PIH	61 (2.2%)	53 (1.8%)	57 (2.0%)	74 (2.6%)	0.220
PTB	179 (6.3%)	187 (6.4%)	187 (6.4%)	238 (8.2%)	0.009
Newborn characteristics					
Sex					
Female	1273 (45.0%)	1360 (46.4%)	1387 (47.8%)	1426 (49.3%)	0.009
Male	1553 (55.0%)	1571 (53.6%)	1517 (52.2%)	1467 (50.7%)	
Birth length (cm)	49.8 ± 1.4	49.9 ± 1.3	49.8 ± 1.5	49.8 ± 1.5	0.297
Birth weight (g)	3346.0 ± 498.1	3353.2 ± 480.1	3339.9 ± 492.5	3330.5 ± 513.3	0.576
Weight for gestational age					
SGA	260 (9.2%)	261 (8.9%)	261 (9.0%)	240 (8.3%)	0.827
AGA	2119 (75.0%)	2221 (75.8%)	2209 (76.1%)	2193 (75.8%)	
LGA	447 (15.8%)	449 (15.3%)	434 (14.9%)	460 (15.9%)	
Laboratory results					
MPV ( fl)	12.1 ± 1.1	11.4 ± 1.0	11.0 ± 1.0	10.5 ± 0.9	< 0.001
RBC (10^12^/L)	4.0 ± 0.3	4.0 ± 0.4	4.0 ± 0.3	4.1 ± 0.4	< 0.001
WBC (10^9^/L)	8.1 ± 2.1	8.6 ± 2.1	8.9 ± 2.2	9.4 ± 2.3	< 0.001
Vitamin B12 (pmol/L)	170.6 ± 76.7	167.0 ± 75.3	156.2 ± 63.1	149.4 ± 66.3	< 0.001
Folic acid (nmol/L)	30.2 ± 14.9	28.3 ± 14.7	25.9 ± 14.0	22.7 ± 13.2	< 0.001
tHcy (μmol/L)	8.5 ± 2.8	8.4 ± 2.6	8.5 ± 2.6	8.7 ± 2.7	0.002
TBA (μmol/L)	5.9 ± 7.5	5.0 ± 4.0	5.1 ± 5.2	5.0 ± 6.6	< 0.001
hs-CRP (mg/L)	2.6 (1.5–4.4)	2.7 (1.5–4.9)	3.0 (1.8–5.2)	3.4 (1.9–5.8)	< 0.001
Total bilirubin (μmol/L)	8.3 ± 3.2	8.1 ± 3.1	7.7 ± 2.7	7.5 ± 2.9	< 0.001
Direct bilirubin (μmol/L)	1.7 ± 1.2	1.6 ± 1.0	1.5 ± 0.9	1.5 ± 0.9	< 0.001
ALT (U/L)	13.9 ± 20.6	11.3 ± 11.8	10.5 ± 8.2	10.4 ± 10.3	< 0.001
AST (U/L)	22.4 ± 27.9	20.0 ± 9.3	19.2 ± 7.1	18.9 ± 9.7	< 0.001
Urea nitrogen (mmol/L)	3.7 ± 1.0	3.6 ± 1.0	3.5 ± 0.9	3.5 ± 0.9	< 0.001
Creatinine (umol/L)	61.4 ± 9.7	60.3 ± 9.7	59.7 ± 8.5	59.2 ± 7.9	< 0.001
Total cholesterol (mmol/L)	6.3 ± 1.2	6.4 ± 1.2	6.4 ± 1.2	6.5 ± 1.2	< 0.001
Triglyceride (mmol/L)	4.0 ± 1.9	3.9 ± 1.8	3.9 ± 1.7	4.0 ± 1.8	0.342
LDL-C (mmol/L)	3.3 ± 0.9	3.4 ± 0.9	3.4 ± 0.9	3.4 ± 0.9	< 0.001
HDL-C (mmol/L)	1.7 ± 0.4	1.7 ± 0.3	1.7 ± 0.3	1.7 ± 0.3	0.121

Data were presented as mean ± SD, median (IQR), and N (%)

*Abbreviations*: *Q* Quartile, *BMI* Body mass index, *BP* Blood pressure, *GDM* Gestational diabetes mellitus, *ICP* Intrahepatic cholestasis of pregnancy, *PE* Pre-eclampsia, *PIH* Pregnancy induced hypertension, *PTB* Preterm birth, *SGA/AGA/LGA* Small/appropriate/large for gestational age, *MPV* Mean platelet volume, *RBC* Red blood cell, *WBC* White blood cell, *tHcy* Total homocysteine, *TBA* Total bile acid, *hs-CRP* High sensitive C-reactive protein, *ALT* Alanine aminotransferase, *AST* Aspartate aminotransferase, *LDL-C* Low density lipoprotein cholesterol, *HDL-C* High density lipoprotein cholesterol, *SD* Standard deviation, *IQR* Interquartile range

^a^115 pregnant women missed height or weight at the time of admission for labor

**Table 2:** The characteristics and laboratory results of pregnant women with and without hyperhomocysteinemia (*n* = 11,553)

	Non-hyperhomocysteinemia (tHcy ≤ 15 μmol/L, *n* = 11,352)	Hyperhomocysteinemia (tHcy > 15 μmol/L, *n* = 201)	*P* value
Maternal characteristics			
Age (years)	28.6 ± 4.4	27.3 ± 5.3	< 0.001
< 35	10,047 (88.5%)	183 (91.0%)	0.262
≥ 35	1305 (11.5%)	18 (9.0%)	
BMI (kg/m^2^)^a^	27.3 ± 3.4	26.7 ± 3.6	0.002
< 25	2796 (24.9%)	62 (31.0%)	0.060
25–29	6173 (54.9%)	108 (54.0%)	
≥ 30	2269 (20.2%)	30 (15.0%)	
Gravidity			
< 3	8083 (71.2%)	128 (63.7%)	0.020
≥ 3	3269 (28.8%)	73 (36.3%)	
Parity			
No child	6827 (60.1%)	108 (53.7%)	0.066
≥ 1 child	4525 (39.9%)	93 (46.3%)	
Gestational age (week)	38.7 ± 1.7	38.5 ± 1.8	0.146
Systolic BP (mmHg)	120.9 ± 12.0	123.1 ± 14.7	0.164
Diastolic BP (mmHg)	74.5 ± 8.2	77.0 ± 9.7	0.001
Assisted reproduction	264 (2.3%)	0 (0.0%)	0.029
Delivery mode			
Vaginal delivery	6525 (57.5%)	117 (58.2%)	0.836
Cesarean section	4827 (42.5%)	84 (41.8%)	
Pregnancy complications			
GDM	961 (8.5%)	4 (2.0%)	0.001
ICP	688 (6.1%)	23 (11.4%)	0.002
PE	369 (3.3%)	25 (12.4%)	< 0.001
PIH	241 (2.1%)	4 (2.0%)	0.897
PTB	767 (6.8%)	24 (11.9%)	0.004
Newborn characteristics			
Sex			
Female	5355 (47.2%)	90 (44.8%)	0.499
Male	5997 (52.8%)	111 (55.2%)	
Birth length (cm)	49.8 ± 1.4	49.4 ± 1.9	< 0.001
Birth weight (g)	3344.7 ± 495.1	3214.0 ± 535.7	< 0.001
Weight for gestational age			
SGA	997 (8.8%)	25 (12.4%)	0.121
AGA	8589 (75.7%)	151 (75.1%)	
LGA	1766 (15.6%)	25 (12.4%)	
Laboratory results			
Platelet (10^9^/L)	202.6 ± 55.4	212.8 ± 62.2	0.031
MPV ( fl)	11.2 ± 1.1	11.3 ± 1.2	0.386
RBC (10^12^/L)	4.0 ± 0.4	3.9 ± 0.4	< 0.001
WBC (10^9^/L)	8.8 ± 2.2	9.1 ± 2.5	0.139
Vitamin B12 (pmol/L)	161.4 ± 69.7	122.6 ± 120.6	< 0.001
Folic acid (nmol/L)	27.0 ± 14.5	12.3 ± 9.0	< 0.001
TBA (μmol/L)	5.2 ± 5.7	8.2 ± 14.7	< 0.001
hs-CRP (mg/L)	3.0 (1.6–5.1)	2.2 (1.1–4.4)	< 0.001
Total bilirubin (μmol/L)	7.9 ± 3.0	8.6 ± 4.8	0.375
Direct bilirubin (μmol/L)	1.6 ± 1.0	2.1 ± 2.4	< 0.001
ALT (U/L)	11.5 ± 13.0	13.7 ± 32.4	< 0.001
AST (U/L)	20.1 ± 15.4	24.1 ± 19.8	0.368
Urea nitrogen (mmol/L)	3.5 ± 0.9	3.8 ± 1.2	< 0.001
Creatinine (umol/L)	60.0 ± 8.8	66.6 ± 15	< 0.001
Total cholesterol (mmol/L)	6.4 ± 1.2	6.4 ± 1.2	0.582
Triglyceride (mmol/L)	4.0 ± 1.8	4.0 ± 2.2	0.960
LDL-C (mmol/L)	3.4 ± 0.9	3.5 ± 0.9	0.042
HDL-C (mmol/L)	1.7 ± 0.3	1.6 ± 0.4	< 0.001

Data were presented as mean ± SD, median (IQR), and N (%)

*Abbreviations*: *tHcy* Total homocysteine, *BMI* Body mass index, *BP* Blood pressure, *GDM* Gestational diabetes mellitus, *ICP* Intrahepatic cholestasis of pregnancy, *PE* Pre-eclampsia, *PIH* Pregnancy induced hypertension, *PTB* Preterm birth, *SGA/AGA/LGA* Small/appropriate/large for gestational age, *MPV* Mean platelet volume, *RBC* Red blood cell, *WBC* White blood cell, *TBA* Total bile acid, *hs-CRP* High sensitive C-reactive protein, *ALT* Alanine aminotransferase, *AST* Aspartate aminotransferase, *LDL-C* Low density lipoprotein cholesterol, *HDL-C* High density lipoprotein cholesterol, *SD* Standard deviation, *IQR* Interquartile range

^a^115 pregnant women missed height or weight at the time of admission for labor

### Associations between PC, tHcy and pregnancy complications

The associations of the prevalence of pregnancy complications with PC and tHcy level were investigated in unadjusted and adjusted logistic regression models (Table 3). Total participants were divided into four groups according to the quartiles of PC and tHcy level, respectively. Compared with the women in Q1 of platelet, women in Q3 and Q4 had substantially lower risks of GDM and ICP. After adjusted for maternal clinical characteristics and relative laboratory results, women in Q4 had still a 24% decreased risks of GDM (OR: 0.76; 95% CI: 0.62, 0.94), and a 32% decreased risks of ICP (OR: 0.68; 95% CI: 0.53, 0.87), respectively. On the contrary, adjusted models showed that women in Q4 of tHcy (> 9.38 μmol/L) had significantly increased risks of ICP, PE, and PIH by 1.9-fold (95% CI: 1.48, 2.45), 2.8-fold (95% CI: 1.95, 3.90), and 2.0-fold (95% CI: 1.31, 2.99), respectively, when compared to those in Q1. In addition, when compared to non-hyperhomocysteinemia women, hyperhomocysteinemia women had higher risks of ICP (OR: 1.69; 95% CI: 1.03, 2.78) and PE (OR: 5.09; 95% CI: 3.05, 8.47) in adjusted models.

**Table 3:** ORs and 95% CIs for pregnancy complications with the categories of maternal platelet count and tHcy level

	GDM		ICP		PE		PIH	
	OR (95%CI)	*P* value	OR (95%CI)	*P* value	OR (95%CI)	*P* value	OR (95%CI)	*P* value
Unadjusted model								
Platelet count (10^9^/L)								
Q1 (< 164)	Ref		Ref		Ref		Ref	
Q2 (164–198)	0.77 (0.65, 0.92)	0.004	0.61 (0.50, 0.75)	< 0.001	0.71 (0.54, 0.95)	0.019	0.78 (0.54, 1.14)	0.203
Q3 (199–236)	0.69 (0.57, 0.83)	< 0.001	0.60 (0.49, 0.74)	< 0.001	0.63 (0.47, 0.84)	0.002	0.84 (0.59, 1.22)	0.365
Q4 (> 236)	0.59 (0.49, 0.71)	< 0.001	0.49 (0.40, 0.61)	< 0.001	0.84 (0.65, 1.11)	0.219	1.09 (0.77, 1.54)	0.618
*P* for trend		< 0.001		< 0.001		0.225		0.442
tHcy level (μmol/L)								
Q1 (< 7.07)	Ref		Ref		Ref		Ref	
Q2 (7.07–8.10)	1.08 (0.90, 1.30)	0.413	1.46 (1.15, 1.87)	0.002	1.46 (1.05, 2.04)	0.025	1.32 (0.90, 1.95)	0.153
Q3 (8.11–9.38)	1.10 (0.92, 1.32)	0.309	1.48 (1.17, 1.89)	0.001	1.78 (1.29, 2.46)	< 0.001	1.44 (0.99, 2.11)	0.057
Q4 (> 9.38)	0.84 (0.69, 1.02)	0.072	2.29 (1.83, 2.86)	< 0.001	2.50 (1.84, 3.39)	< 0.001	1.66 (1.14, 2.40)	0.008
*P* for trend		0.067		< 0.001		< 0.001		0.008
tHcy ≤ 15	Ref		Ref		Ref		Ref	
tHcy > 15	0.26 (0.10, 0.71)	0.008	2.10 (1.35, 3.29)	0.001	4.26 (2.76, 6.60)	< 0.001	1.04 (0.38, 2.84)	0.932
Adjusted model								
Platelet count (10^9^/L)^a^								
Q1 (< 164)	Ref		Ref		Ref		Ref	
Q2 (164–198)	0.85 (0.71, 1.03)	0.098	0.76 (0.61, 0.95)	0.016	0.81 (0.59, 1.10)	0.179	0.72 (0.49, 1.07)	0.103
Q3 (199–236)	0.85 (0.70, 1.04)	0.118	0.84 (0.67, 1.05)	0.122	0.72 (0.52, 0.99)	0.046	0.75 (0.51, 1.10)	0.142
Q4 (> 236)	0.76 (0.62, 0.94)	0.012	0.68 (0.53, 0.87)	0.002	0.92 (0.67, 1.27)	0.614	0.87 (0.60, 1.28)	0.483
*P* for trend		0.016		0.005		0.608		0.663
tHcy level (μmol/L)^b^								
Q1 (< 7.07)	Ref		Ref		Ref		Ref	
Q2 (7.07–8.10)	1.16 (0.95, 1.41)	0.139	1.34 (1.04, 1.73)	0.023	1.50 (1.06, 2.14)	0.023	1.42 (0.95, 2.12)	0.090
Q3 (8.11–9.38)	1.33 (1.09, 1.61)	0.005	1.34 (1.03, 1.73)	0.027	1.90 (1.34, 2.68)	< 0.001	1.57 (1.05, 2.35)	0.028
Q4 (> 9.38)	1.22 (0.98, 1.52)	0.074	1.90 (1.48, 2.45)	< 0.001	2.76 (1.95, 3.90)	< 0.001	1.98 (1.31, 2.99)	0.001
*P* for trend		0.040				< 0.001		0.001
≤ 15 μmol/L	Ref		Ref		Ref		Ref	
> 15 μmol/L	0.32 (0.10, 1.01)	0.053	1.69 (1.03, 2.78)	0.038	5.09 (3.05, 8.47)	< 0.001	1.20 (0.42, 3.42)	0.734

Adjusted for age, gravidity, parity, BMI, assisted reproduction, neonatal sex, RBC and WBC count, hs-CRP, total bilirubin, direct bilirubin, ALT, AST, urea nitrogen, creatinine, HDL-C, LDL-C, total cholesterol, triglyceride, vitamin B12, folic acid and TBA levels

*Abbreviations*: *GDM* Gestational diabetes mellitus, *ICP* Intrahepatic cholestasis of pregnancy, *PE* Pre-eclampsia, *PIH* Pregnancy induced hypertension, *OR* Odds ratio, *CI* Confidence interval, *Q* Quartile, *BMI* Body mass index, *RBC* Red blood cell, *WBC* White blood cell, *TBA* Total bile acid, *hs-CRP* High sensitive C-reactive protein, *ALT* Alanine aminotransferase, *AST* Aspartate aminotransferase, *HDL-C* High density lipoprotein cholesterol, *LDL-C* Low density lipoprotein cholesterol, *tHcy* total homocysteine

^a^additionally adjusted for tHcy level

^b^additionally adjusted for platelet count

### Joint influence of PC and tHcy on pregnancy complications

Table 4 quantifies the modification effects of platelet count on associations of hyperhomocysteinemia with GDM, ICP, and PE. For those with hyperhomocysteinemia (compared with non-hyperhomocysteinemia group), the ICP prevalence increased from 8.4% to 28.6% in the low PC quartile (Q1), representing an absolute risk increment of 20.2% and a relative risk increment of 3.3-fold in the adjusted model (OR: 3.34; 95% CI: 1.55, 7.17; *P* = 0.002). In contrast, a moderate reduction in the ICP risk was observed for the high PC quartiles (Q2– Q4) (OR: 0.70; 95% CI: 0.34, 1.41; *P* = 0.313). A interaction test between PC and hyperhomocysteinemia on ICP was statistically significant (*P* for interaction = 0.014). With regards to the GDM risk, a significant interaction between PC and hyperhomocysteinemia was observed only in the crude models (*P* for interaction = 0.042). The GDM prevalence decreased from 10.5% in the non-hyperhomocysteinemia women with PC Q1 to 0.6% in the hyperhomocysteinemia women with PC Q2–Q4, suggesting an absolute risk decline of 9.9% and a relative risk reduction of 95% in the unadjusted model (OR: 0.05; 95% CI: 0.01, 0.39; *P* = 0.004). However, the interaction test did not remain significant in the adjusted model (*P* for interaction = 0.09).

**Table 4:** Effect modification of platelet on associations between hyperhomocysteinemia and pregnancy complications

	Non-hyperhomocysteinemia		Hyperhomocysteinemia		Crude		*P* value^a^ for Interaction	Adjusted^b^		*P* value^a^ for Interaction
	Total	patients (%)	Total	patients (%)	OR (95%CI)	*P* value		OR (95%CI)	*P* value	
GDM										
Platelet Q1	2783	292 (10.5%)	42	3 (7.1%)	0.66 (0.20, 2.14)	0.484	Ref	0.77 (0.20, 3.04)	0.711	Ref
Platelet Q2–Q4	8569	669 (7.8%)	159	1 (0.6%)	0.05 (0.01, 0.39)	0.004	0.042	0.09 (0.01, 0.67)	0.018	0.090
Platelet Q2	2886	251 (8.7%)	45	1 (2.2%)	0.19 (0.03, 1.41)	0.105		0.37 (0.05, 2.76)	0.333	
Platelet Q3	2856	225 (7.9%)	48	0 (0.0%)	—	—		—	—	
Platelet Q4	2827	193 (6.8%)	66	0 (0.0%)	—	—		—	—	
ICP										
Platelet Q1	2783	234 (8.4%)	42	12 (28.6%)	4.36 (2.20, 8.62)	< 0.001	Ref	3.34 (1.55, 7.17)	0.002	Ref
Platelet Q2–Q4	8569	454 (5.3%)	159	11 (6.9%)	0.81 (0.43, 1.52)	0.509	0.012	0.70 (0.34, 1.41)	0.313	0.014
Platelet Q2	2886	164 (5.7%)	45	2 (4.4%)	0.51 (0.12, 2.10)	0.349		0.47 (0.11, 1.98)	0.303	
Platelet Q3	2856	160 (5.6%)	48	4 (8.3%)	0.99 (0.35, 2.78)	0.985		0.81 (0.24, 2.68)	0.726	
Platelet Q4	2827	130 (4.6%)	66	6 (9.1%)	0.89 (0.36, 2.24)	0.810		0.78 (0.28, 2.20)	0.638	
PE										
Platelet Q1	2783	111 (4.0%)	42	4 (9.5%)	2.53 (0.89, 7.22)	0.082	Ref	2.75 (0.78, 9.68)	0.116	Ref
Platelet Q2–Q4	8569	258 (3.0%)	159	21 (13.2%)	3.66 (2.23, 6.02)	< 0.001	0.237	3.67 (2.07, 6.49)	< 0.001	0.312
Platelet Q2	2886	82 (2.8%)	45	9 (20.0%)	6.02 (2.83, 12.80)	< 0.001		8.45 (3.76, 19.02)	< 0.001	
Platelet Q3	2856	74 (2.6%)	48	6 (12.5%)	3.44 (1.43, 8.26)	0.006		2.72 (0.98, 7.56)	0.056	
Platelet Q4	2827	102 (3.6%)	66	6 (9.1%)	2.41 (1.02, 5.69)	0.045		2.10 (0.80, 5.52)	0.133	

*Abbreviations*: *OR* Odds ratio, *CI* Confidence interval, *Q* Quartile, *GDM* Gestational diabetes mellitus, *ICP* Intrahepatic cholestasis of pregnancy, *PE* Pre-eclampsia, *BMI* Body mass index, *RBC* Red blood cell, *WBC* White blood cell, *TBA* Total bile acid, *hs-CRP* High sensitive C-reactive protein, *ALT* Alanine aminotransferase, *AST* Aspartate aminotransferase, *HDL-C* High density lipoprotein cholesterol, *LDL-C* Low density lipoprotein cholesterol, *tHcy* Total homocysteine

^a^Two-way interaction test for platelet (Q1 vs. Q2–Q4) and tHcy ( non-hyperhomocysteinemia vs. Hyperhomocysteinemia) on pregnancy complications

^b^Adjusted for age, gravidity, parity, BMI, assisted reproduction, neonatal sex, RBC and WBC count, hs-CRP, total bilirubin, direct bilirubin, ALT, AST, urea nitrogen, creatinine, HDL-C, LDL-C, total cholesterol, triglyceride, vitamin B12, folic acid, and TBA levels

### Subgroup analysis by important covariates

To further verify that the results in Table 4 are steady to potential confounding factors, a stratified analysis of subgroups categorized by major covariates was performed, including age, BMI, parity, folic acid and vitamin B12 levels. All analysis was adjusted for age, gravidity, parity, BMI, assisted reproduction, RBC and WBC count, hs-CRP, total bilirubin, direct bilirubin, ALT, AST, urea nitrogen, creatinine, HDL-C, LDL-C, total cholesterol, triglyceride, folic acid, and vitamin B12 levels, except for the covariate that was stratified. Table 5 reveals a highly consistent model: among participants with PC Q1, regardless of subgroup, hyperhomocysteinemia resulted in a significant increment in ICP risk, with ORs ranging from 1.64 to 7.66. On the contrary, among women with PC Q2–Q4, the efficacy of hyperhomocysteinemia was greatly attenuated, with ORs ranging from 0.39 to 1.35.

**Table 5:** Subgroup analysis of the effect modification of platelet on associations between hyperhomocysteinemia and ICP

	Non-hyperhomocysteinemia		Hyperhomocysteinemia		Crude		*P* value^a^ for Interaction	Adjusted^b^		*P* value^a^ for Interaction
	Total	patients (%)	Total	patients (%)	OR (95%CI)	*P* value		OR (95%CI)	*P* value	
Platelet Q1										
Age (years)										
< 35	2402	195 (8.1%)	37	11 (29.7%)	4.79 (2.33, 9.84)	< 0.001	Ref	4.15 (1.79, 9.57)	< 0.001	Ref
≥ 35	381	39 (10.2%)	5	1 (20.0%)	2.83 (0.31, 25.44)	0.353	0.488	3.14 (0.31, 31.63)	0.331	0.615
BMI (kg/m^2^)^a^										
< 25	758	66 (8.7%)	19	6 (31.6%)	4.84 (1.78, 13.15)	0.002	Ref	4.95 (1.58, 15.49)	0.006	Ref
25–29	1526	128 (8.4%)	19	6 (31.6%)	4.84 (1.78, 13.15)	0.002		4.93 (1.57, 15.43)	0.006	
≥ 30	474	36 (7.6%)	4	0 (0.0%)	—	—	0.281	—	—	0.217
Parity										
No child	1586	135 (8.5%)	22	8 (36.4%)	6.14 (2.53, 14.90)	< 0.001	Ref	4.63 (1.60, 13.38)	0.005	Ref
≥ 1 child	1197	99 (8.3%)	20	4 (20.0%)	2.69 (0.89, 8.15)	0.081	0.265	3.93 (1.13, 13.68)	0.032	0.671
Folic acid (nmol/L)										
< 10	1134	102 (9.0%)	35	9 (25.7%)	1.96 (0.47, 8.14)	0.355	Ref	1.64 (0.37, 7.26)	0.518	Ref
≥ 10	1649	132 (8.0%)	7	3 (42.9%)	3.40 (1.23, 9.37)	0.018	0.227	3.27 (1.05, 10.23)	0.042	0.121
Vitamin B12 (pmol/L)										
< 150	530	42 (7.9%)	21	4 (19.0%)	2.52 (0.94, 6.77)	0.067	Ref	2.45 (0.85, 7.11)	0.098	Ref
≥ 150	2253	192 (8.5%)	21	8 (38.1%)	11.59 (3.98, 33.70)	< 0.001	0.052	7.66 (2.25, 26.06)	0.001	0.138
Platelet Q2–Q4										
Age (years)										
< 35	7645	405 (5.3%)	146	11 (7.5%)	1.46 (0.78, 2.71)	0.236	Ref	0.95 (0.47, 1.93)	0.893	Ref
≥ 35	924	49 (5.3%)	13	0 (0.0%)	—	—	0.165	—	—	0.247
BMI (kg/m^2^)^a115^										
< 25	2037	129 (6.3%)	43	5 (11.6%)	1.95 (0.75, 5.03)	0.169	Ref	1.35 (0.47, 3.91)	0.575	Ref
25–30	4648	230 (4.9%)	89	3 (3.4%)	0.52 (0.16, 1.65)	0.265		0.39 (0.11, 1.30)	0.125	
> 30	1795	91 (5.1%)	26	3 (11.5%)	1.93 (0.57, 6.51)	0.290	0.218	1.30 (0.31, 5.49)	0.717	0.316
Parity										
No child	5242	284 (5.4%)	86	7 (8.1%)	1.55 (0.71, 3.38)	0.274	Ref	1.16 (0.49, 2.72)	0.739	Ref
≥ 1 child	3327	170 (5.1%)	73	4 (5.5%)	1.01 (0.37, 2.79)	0.982	0.576	0.65 (0.20, 2.13)	0.474	0.378
Folic acid (nmol/L)^b4^										
< 10	4587	276 (6.0%)	147	10 (6.8%)	0.92 (0.38, 2.24)	0.851	Ref	0.76 (0.29, 1.99)	0.571	Ref
≥ 10	3978	178 (4.5%)	12	1 (8.3%)	0.92 (0.35, 2.43)	0.874	0.542	0.59 (0.20, 1.79)	0.354	0.739
Vitamin B12 (pmol/L)^c2^										
< 150	1992	101 (5.1%)	106	4 (3.8%)	1.33 (0.67, 2.64)	0.419	Ref	0.98 (0.47, 2.04)	0.952	Ref
≥ 150	6575	353 (5.4%)	53	7 (13.2%)	2.13 (0.49, 9.24)	0.312	0.701	0.44 (0.05, 3.88)	0.463	0.436

Adjusted for age, gravidity, parity, BMI, assisted reproduction, neonatal sex, RBC and WBC count, hs-CRP, total bilirubin, direct bilirubin, ALT, AST, urea nitrogen, creatinine, HDL-C, LDL-C, total cholesterol, triglyceride, folic acid, vitamin B12, and TBA levels, except for the covariate that was stratified. These stratified variables, including age, BMI, parity, folic acid, vitamin B12 did not significantly modified the effect of hyperhomocysteinemia on ICP incidence; the* P* values for all interaction tests were > 0.05

*Abbreviations*: *ICP* Intrahepatic cholestasis of pregnancy, *OR* Odds ratio, *CI* Confidence interval, *Q* Quartile, *BMI* Body mass index, *RBC* Red blood cell, *WBC* White blood cell, *TBA* Total bile acid, *hs-CRP* High sensitive C-reactive protein, *ALT* Alanine aminotransferase, *AST* Aspartate aminotransferase, *HDL-C* High density lipoprotein cholesterol, *LDL-C* Low density lipoprotein cholesterol, *tHcy* Total homocysteine

## Discussion

### Main findings

So far as we know, this is the largest hospital-based study to report retrospective associations of pregnancy complications with platelet count and tHcy levels in a Chinese population. We observed negative associations of PC quartiles with GDM and ICP prevalence, which was 10.4% and 8.7% in Q1, and decreased to 8.6% and 5.7%, 7.7% and 5.6%, 6.7% and 4.7, in Q2, Q3, and Q4, respectively. The highest rate of GDM (10.5%) was observed in the low PC Q1 and low tHcy (tHcy ≤ 15 μmol/L) subgroup, whereas the lowest rate (0.6%) was observed in the high PC (Q2–Q4) and high tHcy subgroup. We also found a remarkable differences in the efficacy of hyperhomocysteinemia across subgroups. The greatest risk increment in ICP for those with hyperhomocysteinemia was observed in the low PC Q1 group (from 8.4% to 28.6%), a risk increment of 3.3-fold (OR: 3.34; 95% CI: 1.55, 7.17; *P* = 0.002). In contrast, hyperhomocysteinemia had no effect on ICP in the high PC group. Taken together, our findings suggest that pregnant Chinese women with both low PC and hyperhomocysteinemia are at highest risk for ICP, while those with high PC and hyperhomocysteinemia are at lowest risk for GDM. These results, if confirmed, could help identify those pregnant women who are at high risk of ICP and low risk of GDM.

### Platelet and pregnancy complications

Previous studies have evaluated potential platelet-associated alterations in pregnancy complications. A prospective case–control study from Turkey (40 cases of ICP and 40 controls) found that MPV values were higher in patients with ICP and positively associated with D-dimer in all participants during the third trimester of pregnancy [24]. Results from another Turkish prospective case–control study (117 cases of ICP and 100 controls) reported that patients with ICP had significantly higher MPV and platelet distribution width (PDW) values and an elevated MPV was related to PTB [25]. The third retrospective case–control study from Turkey (84 cases of ICP and145 age-matched controls) further indicated that MPV can be used as an indicator of disease severity in patients with ICP [26]. In addition, Silva et al. in USA expanded the previous studies on association between platelet indices and ICP in both early and late pregnancy from 33 patients with ICP and 33 controls matched for age, parity, and race [27]. However, they found no significant differences in platelet indices (MPV/PDW/PC) between ICP and control in the first trimester and between mild and severe ICP in the third trimester. In the present cohort study, we found that women with ICP had a higher MPV (11.6 vs. 11.1 fl, *P* < 0.001) and a lower PC (187 vs. 200 10^9^/L, *P* < 0.001), when compared to women without pregnancy complications, and there were significant decreased trends in the prevalence of ICP from PC Q1 to Q4 (8.7% vs. 5.7% vs. 5.6% vs.4.7%, *P* < 0.001). Similar findings were observed in women with GDM (MPV: 11.2 vs. 11.1 fl, *P* = 0.003; PC: 191 vs. 200 10^9^/L, *P* < 0.001; 10.4% vs. 8.6% vs. 7.7% vs.6.7%, *P* < 0.001). In addition, our study revealed there is a significant increment in MPV and a significant decrement in PC among women with PE during late pregnancy (MPV: 11.4 vs. 11.1 fl, *P* = 0.003; PC: 194 vs. 200 10^9^/L, *P* < 0.001), which is agreement with the findings from a recent meta-analysis [23]. Our results suggest that pregnant women with late pregnancy complications such as GDM, ICP, and PE exhibit signs of low-grade activation in the coagulation system, as evidenced by changes in platelet morphology and function. [23]. Our study also found that women with PIH had a significant increase only in MPV but not in PC when compared to women without pregnancy complications (MPV: 11.3 vs. 11.1 fl, *P* = 0.004; PC: 202 vs. 200 10^9^/L, *P* = 0.336). This is perhaps because PE and PIH are manifestations of different severity of the same pathophysiology [37].

### Hyperhomocysteinemia and pregnancy complications

Elevated tHcy level may lead to DNA damage and endothelial dysfunction by increasing oxidative stress and inflammatory response, and has been associated with numerous diseases [38]. Hyperhomocysteinemia exerts a wide range of pathological effects on maternal endothelial injury and placental vascular dysfunction by increasing the release of inflammatory cytokines from vascular endothelial cells and promoting the proliferation of vascular smooth cells. It has inconsistent associations with placenta-mediated complications [28]. In our study, pregnant women with hyperhomocysteinemia had a 5.1-fold greater risk of PE (OR: 5.09; 95% CI: 3.0, 8.4; *P* < 0.001) and a 1.7-fold increased risk of ICP (OR: 1.69; 95% CI: 1.03, 2.78; *P* = 0.038) compared to those with non-hyperhomocysteinemia. Our results are in agreement with a recent meta-analysis that revealed an increased risk of PE associated with elevated homocysteine levels [39]. However, other studies found no association [40, 41]. In addition, we showed that pregnant women with hyperhomocysteinemia had a significantly lower prevalence of GDM (2.0% vs. 8.5%, *P* = 0.001) compared to those with non-hyperhomocysteinemia, which is similar with the previous study by López-Quesada et al., who reported that higher tHcy levels were associated with decreased odds of GDM [42]. However, a meta-analysis and a recent case–control study showed that GDM women exhibited elevated tHcy levels, and GDM risk was 1.79-fold higher in women with high tHcy (≥ 7.29 μmol/L) relative to those with low tHcy (< 5.75 μmol/L) [43, 44]. In summary, although a number of studies have investigated the associations of pregnancy complications with tHcy levels and PC, the results remain inconclusive and inconsistent, especially for GDM and PE. The reason for discrepancy could be the difference in population frequency of the MTHFR (677 C > T) polymorphism, and in cut-off criteria of hyperhomocysteinemia in various studies. MTHFR polymorphism might contribute to moderate elevation of tHcy level [45]. Different cut-off values, including quartiles, tertiles, percentiles, and tHcy > 10 or 15 μmol/L were used to define hyperhomocysteinemia in previous studies [28]. In addition, this discrepancy could also be explained by epidemiological study designs, sample sizes, geographical location and ethnicity, the timing of sample collection, gestational weeks of study participants, and adjustment for confounding factors.

### Possible mechanism linkages

The mechanism by which maternal low PC and high tHcy could jointly increase ICP prevalence remains unknown. Over the past 40 years, there is a growing evidence to document the role of hyperhomocysteinemia in causing vascular damage and promote thrombosis that triggers a coagulation process contributing to platelet activation and consumption [46]. Our results appear to be in agreement with the previous study by Kong et al., who reported a joint effect of high tHcy and low PC on increased first stroke risk [47]. Our findings support one speculation that maternal/placental endothelial damage and thrombosis were involved in the pathogenesis of ICP [9]. The elevated MPV found in women with low PC group (Q1) in the present study further supports this speculation, since the large platelet is more reactive, production of prothrombotic factors, and aggregation [29]. The findings on a joint effect of high tHcy and low PC on elevated ICP prevalence also support that a combination of platelet and tHcy could also be a marker for endothelial injury and thrombosis [47].

### Strengths and limitations

This study contribute new information to the literature on the adverse effect of hyperhomocysteinemia on ICP, which could be further modified by platelet count. This large hospital-based observational cohort study ensure us to correct various important confounders, including blood lipids and hs-CRP levels, hepatic and renal function. Importantly, we adjusted the status of folic acid and vitamin B12. Finally, the present study is also one of the few to evaluate the relation between platelet count and ICP during prenatal period. However, some limitations of the present study should also be mentioned. First, this is a single-center, post-hoc analysis that can prove an association but not a causal relationship and its generalizability for other centers needs to be confirmed. Second, although multivariate-adjusted logistic regression models were performed, some uncollected data or undetected variables might affect the prevalence of pregnancy complications. For example, we did not have data on whether the participants underwent sex-selection abortions during their previous pregnancy, which may affect their platelet counts and overall health status [48, 49]. Hence, the influence of residual confounders should be considered. Third, this was a retrospective cohort study, which may introduce bias. In addition, the present study did not investigate the underlying mechanisms for the observed associations, which may necessitate additional research.

## Conclusion

Among 11, 553 Chinese pregnant women in a 3-A-Class Specialized Hospital, we revealed that the subgroup with low PC and high tHcy level at the time of admission for labor has the highest risk of ICP, while the other subgroup with high PC and high tHcy level has the lowest risk of GDM. These finding, if confirmed, enable us to identify these individuals who are at high risk of ICP or low risk of GDM with a combination of platelet count and tHcy level (both tests are easy to get). Our findings would be helpful in the screening and management of pregnancy complications in Chinese populations.

## Data Availability

The datasets used and/or analyzed during the current study are available from the corresponding author on reasonable request.

## Abbreviations

GDM: Gestational diabetes mellitus
ICP: Intrahepatic cholestasis of pregnancy
PE: Pre-eclampsia
PIH: Pregnancy induced hypertension
SGA/AGA/LGA: Small/appropriate/large for gestational age
PTB: Preterm birth
BMI: Body mass index
BP: Blood pressure
Q: Quartile
OR: Odds ratio
CI: Confidence interval
SD: Standard deviation
MPV: Mean platelet volume
PC: Platelet count
PDW: Platelet distribution width
RBC: Red blood cell
WBC: White blood cell
tHcy: Total homocysteine
hs-CRP: High sensitive C-reactive protein
ALT: Alanine aminotransferase
AST: Aspartate aminotransferase
LDL-C: Low density lipoprotein cholesterol; HDL-C, high density lipoprotein cholesterol
